# A Manual of General Anatomy, by F. J. Meckel, Professor of Anatomy at Halle, &c.

**Published:** 1837-10

**Authors:** 


ART. IX.—*A Manual of General Anatomy, by* F. J. MECKEL, Professor of Anatomy at Halle, &c. *Translated from German into French, with additional Notes, by* A. J. L. JOURDAN *and* G. BRESCHET. *Translated from the French, with Notes, by* A. S. DOANE, A.M. M.D. *and others.—London,* 1837. 8vo. pp. 421.

THE original of this work has now been more than ten or a dozen years before the public, and has gained so high a character that it requires but few comments of ours to recommend it to the attention of the profession. We have always objected to a work being translated through two languages, because it almost invariably happens that certain portions of the author's meaning are lost, or not sufficiently explained; and if, in the first translation, either of these errors be committed, they frequently become doubled, and perhaps trebled, when the work is retranslated into another language. The translators themselves, in the present instance, appear to be fully aware of this; and we cannot but regret that, instead of employing their time in translating Jourdan, they had not devoted it to the study of the noble original language. We find many inaccuracies in the text thus produced; and, on looking over the notes, it will be seen that they are almost entirely French, and that the standard of information which is professed to be given in them is not brought up nearly to the present day. This is a great defect. The quantity of information which has been accumulating through the last five or six years is much too valuable and too abundant to be overlooked in a translation which professes to supply commentaries upon the original. Our criticism is more called for on the present occasion, because we perceive, from a notice attached to the title-page of the present work, that the same authors are about to produce a translation of Meckel's "Descriptive and Pathological Anatomy," derived, like the present, from the French.—We need allude to scarcely more than one instance out of many which we might cite to prove the necessity of a careful attention to the information contained in the notes in illustration of doubtful or incorrect views in the original. At page 223 it is stated, on the authority of Cuvier's "Anatomie Comparée," vol. ii., that in many worms only a single cord exists, which extends throughout the whole of the body, but without giving off any nerves. Now, upon the truth of this statement, much of a great theory of development depends. Although it is the belief of many anatomists, we ourselves have always doubted the correctness of the alleged fact, on analogical principles; and Mr. Swan* has recently shown that it is incorrect with regard to Strongylus gigas. We have the strongest belief that this will also be found to be the case in every other instance.

In hopes that Dr. Doane will take our advice and study German before he publishes any more translations of German works, we are bound to say that, with all its defects, the present little volume will be useful to the English student. Its conciseness, and the great mass of interesting and important matter contained in it, cannot fail to render it a valuable acquisition to all who are ignorant of the languages out of which it is translated.

* Cyclopædia of Practical and Comparative Anatomy, Part X. vol. ii. p. 130.

